# Prognostic impact of fluorescent lymphography on gastric cancer

**DOI:** 10.1097/JS9.0000000000000572

**Published:** 2023-06-21

**Authors:** Sung Hyun Park, Ki-Yoon Kim, Minah Cho, Yoo Min Kim, Hyoung-Il Kim, Woo Jin Hyung

**Affiliations:** a Department of Surgery, Yonsei University College of Medicine; b Gastric Cancer Center, Yonsei Cancer Center, Yonsei University Health System, Seoul, Republic of Korea

**Keywords:** gastric cancer, lymphadenectomy, prognosis, near-infrared imaging, fluorescent lymphography

## Abstract

**Background:**

Fluorescent lymphography-guided lymphadenectomy (FL) for gastric cancer is gaining popularity. However, its impact on prognosis is not known. This study aimed to assess the prognostic impact of FL in gastric cancer patients.

**Materials and methods:**

This study retrospectively analyzed 5678 gastric cancer patients who underwent gastrectomy from 2013 to 2017. The survival was compared between the FLFL group and the conventional lymphadenectomy (non-FL group) using 1:1 propensity score matching after exclusion. Patients in the FL group underwent gastrectomy with systematic lymphadenectomy after endoscopic peritumoral injection of indocyanine green the day before surgery.

**Results:**

After propensity score matching, the FL and non-FL groups each had 1064 patients with similar demographic and clinicopathological characteristics. All matched variables had a standardized mean difference under 0.1. The FL group showed a significantly higher number of retrieved lymph nodes (56.2±20.1) than the non-FL group (46.2±18.2, *P*<0.001). The FL group also had more stage III patients (*P=*0.044) than the non-FL group. The FL group demonstrated higher overall survival (*P=*0.038) and relapse-free survival (*P=*0.036) in stage III compared with the non-FL group. However, no significant differences in overall and relapse-free survival were observed between the two groups for stages I (*P=*0.420 and *P=*0.120, respectively) and II (*P=*0.200 and *P=*0.280, respectively).

**Conclusion:**

FL demonstrated a higher survival in stage III gastric cancer patients by the more accurate staging resulting from larger lymph node retrieval. Thus, given its potential to improve prognostication by enhancing staging accuracy, it is recommended as an option to consider the use of FL in clinical practice.

## Introduction

Highlights

- This study assessed the prognostic impact of fluorescent lymphography-guided lymphadenectomy in gastric cancer patients.
- The fluorescent lymphadenectomy group showed a significantly higher number of retrieved lymph nodes, which resulted in more advanced stages.
- Fluorescent lymphography-guided lymphadenectomy provides a higher survival of stage III gastric cancer patients by the more accurate staging

Gastric cancer is one of the leading causes of cancer-related mortality worldwide^1–4^. Radical gastrectomy with systematic lymphadenectomy offers the best opportunity to cure patients with localized gastric cancers^5,6^. The extent of lymphadenectomy in gastric cancer is recommended based on the probability of lymph node (LN) metastasis for each station and information about the lymphatic drainage pattern related to the tumour location and depth of invasion^7,8^. Additionally, obtaining a sufficient number of retrieved LNs leads to adequate nodal staging in the current tumour-node-metastasis staging system, which has also been associated with better survival^9,10^.

Thus, using vital dyes such as patent blue or carbon nanoparticles during radical gastrectomy for thorough lymphadenectomy has been reported before the advent of indocyanine green (ICG)^11,12^. These vital dyes facilitated the visualization of lymphatics draining from the tumour, thus aiding in an adequate and effective lymphadenectomy. Using carbon nanoparticles during lymphadenectomy in radical gastrectomy had shown potential in harvesting more LNs and aiding the detection of metastatic LNs^13,14^.

Recent advances in fluorescent-based lymphatic imaging using near-infrared technology have further improved the visualization of the lymphatic system and LNs draining from the primary tumour^15–18^. ICG fluorescent lymphography using near-infrared imaging enables easier and more thorough lymphadenectomy during radical gastrectomy^17,19^. Moreover, ICG FL increased LN retrieval and metastatic LN detection with high sensitivity and low negative predictive value^19^. However, the oncological impact of ICG fluorescent lymphography-guided lymphadenectomy (FL) on gastric cancer patients has not been studied. Therefore, this study aimed to assess the impact of ICG FL on the prognosis of gastric cancer patients by comparing the long-term oncological outcomes with conventional lymphadenectomy (non-FL).

## Materials and methods

### Patients

A retrospective review of a prospectively collected gastric cancer database identified 5678 patients who underwent gastrectomy between 2013 and 2017. Patient and tumour characteristics, surgical information, and pathological features were prospectively collected in the database. Patients were grouped into a FL group or a non-FL group. The inclusion criteria were (1) histologically proven gastric adenocarcinoma, (2) a single primary lesion, and (3) a clinical stage of T1-4aN0-3M0. The exclusion criteria were (1) open surgery and open conversion from laparoscopic or robotic gastrectomy, (2) preoperative chemotherapy or radiation therapy for current gastric cancer, (3) R1 or R2 resection, (4) pylorus-preserving gastrectomy or less than D1+ dissection, (5) open conversion from laparoscopic or robotic gastrectomy, (5) incomplete information on clinical or pathological features, or (6) surgical mortality. The reason open surgery was excluded from the study was due to the absence of a fluorescent imaging device during the study period. ICG was not injected in patients scheduled for open gastrectomy. Also, patients who received neoadjuvant chemotherapy or radiotherapy were excluded due to potential changes in tumour characteristics and lymphatic drainage patterns, which could influence the outcomes of ICG injection and fluorescent-guided lymphadenectomy^20^. Pylorus-preserving gastrectomy was excluded because if fluorescent-positive infrapyloric lymph nodes are observed when using fluorescent lymphography, the resection extent should be changed.

This study was conducted in accordance with the STROCSS criteria, Supplemental Digital Content 1, http://links.lww.com/JS9/A737 and approved by the Institutional Review Board (4-2020-0082)^21^. Informed consent was waived due to the retrospective nature of the study.

### Endoscopic ICG injection

Early in the study, ICG (Dongindang Pharmaceutical) was diluted using distilled water to a concentration of 1.25 mg/ml. At four points around the primary tumour, 0.6 ml ICG solution was endoscopically injected into the submucosal layer on the day before surgery during a routine endoscopic preoperative localization process. Thus, a total of 3 mg ICG was used. However, with the advancement in fluorescent imaging devices, the administered amount of ICG was subsequently reduced. The da Vinci Si (Intuitive Surgical) System employs a conventional laparoscopic camera arrangement with an externally placed camera. In contrast, the da Vinci Xi (Intuitive Surgical) System uses an inside-the-patient chip-on-a-tip configuration, resulting in higher sensitivity to fluorescent signals and displaying stronger signals^22^.

As a result of system differences and enhancements in fluorescent imaging sensitivity, the concentration of ICG needed to be optimized. From 2015 onward, ICG was diluted to a concentration of 0.625 mg/ml, and the total amount of ICG used was 1.5 mg, which is half the amount of the previously used. The feasibility of this institutional preoperative peritumoral ICG injection protocol has previously been reported^15–17,19,23^.

### Surgery

When performing laparoscopic surgery, a Pinpoint endoscopic fluorescent imaging system was used. The Firefly mode in a da Vinci Si or Xi surgical system was used when robotic surgery was performed. FL was performed by switching between the general visible light and near-infrared fluorescent views with a simple mode change during both laparoscopic and robotic surgeries. Depending on the location and clinical stage of the lesion, subtotal gastrectomy, total gastrectomy, or proximal gastrectomy was conducted. According to the Korean Practice Guideline for Gastric Cancer, D1+ lymphadenectomy was performed for early gastric cancer patients with any suspicion of LN metastasis, and D2 lymphadenectomy was performed for patients with suspicion of LN metastasis or advanced gastric cancer^7^. Near-infrared imaging was used before, during, and after each LN station dissection to confirm whether any fluorescent LNs remained in the dissected area; if remained, fluorescent tissue was additionally removed. When a fluorescent LN existed outside the originally planned dissection area, additional dissection was performed if it belonged to the D2 area. Areas outside D2, except 14v, were not dissected.

### Postoperative LN harvesting

The surgeon separated LN-containing soft tissue from the specimen obtained after radical gastrectomy and classified LN stations based on the Japanese classification^24^. The surgeon then checked each LN station to determine whether it included LNs containing fluorescent components using *ex vivo* near-infrared imaging. LNs exhibiting ICG fluorescent emission were classified as fluorescent LNs, and LNs without fluorescent were classified as non-fluorescent LNs. Stations containing fluorescent LNs were classified as fluorescent stations, and the others were classified as non-fluorescent stations. All retrieved tissues and LNs were transferred to pathologists, who performed pathological examinations on specimens separated by the stations. Nodal staging evaluation was performed on tissues classified as fluorescent or non-fluorescent. Specimens fixed in paraffin blocks were again examined to confirm the presence of fluorescence in LNs.

### Patient management and follow-up

Based on the Korean practice guideline, S-1 or capecitabine plus oxaliplatin adjuvant chemotherapy was recommended for stage II or III patients after radical gastrectomy^7^. Patients were followed-up until their death or until 31 December 2019. Survival and recurrence status was verified based on both medical records of the hospital and survey data from the National Statistical Office of Korea.

### Statistical analysis

Propensity score matching was used for clinicopathological features to minimize selection bias. Propensity score matching analysis adheres to the EQUATOR (Enhancing the Quality and Transparency of Health Research) network reporting guidelines^25^. Factors that could affect pathological stages or long-term outcomes were matched; in detail, the caliper value was set to 0.2, which is close to 20% of the standard deviation of the logit-transformed propensity scores (0.1799) for 1:1 matching using the nearest method with a none-discard strategy, adjusting for the following factors: patient demographics [age, sex, BMI, and American Society of Anesthesiology (ASA) score], perioperative tumour characteristics (clinical T stage, clinical N stage, differentiation, tumour location, and tumour size), surgical extent (resection extent and LN dissection extent), and operation year^26^. Using the default settings, the seed value was not specified. The distance metric was set to “logit,” and the sampling replacement was set to false. Standardized mean difference and generalized variance ratio were calculated to evaluate the balance between the two groups after propensity score matching. Continuous variables are expressed using the mean and standard deviation or median value and interquartile range. As appropriate, the Student’s *t*-test or the Mann–Whitney U test was used. For categorical variables, the χ^2^ or Fisher’s exact test was used. Survival was evaluated using the Kaplan–Meier method, with the numbers at-risk also presented. The log-rank test was performed to compare the overall survival and relapse-free survival between the two groups. A value of *P* less than 0.05 was considered significant. Statistical analyses of the study were conducted using SPSS statistical software for Windows, version 25 (SPSS) or R packages (Survival, Matchit and Cobalt, version 4.0.4, 2021; R Foundation for Statistical Computing).

## Results

### Patient demographics and propensity score matching

**Figure 1:**
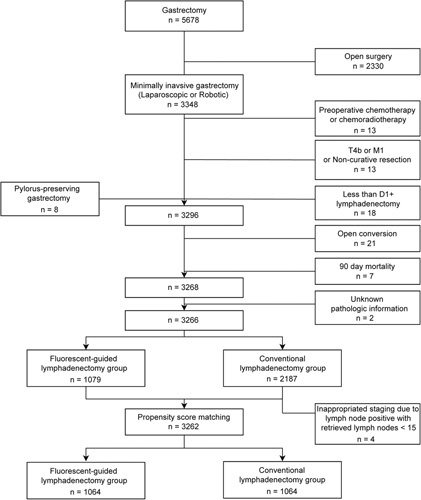
Study profile.

As shown in Fig. 1, a total of 3266 patients were included in the analysis after the exclusion process. In total, there were 1079 patients in the FL group and 2187 patients in the non-FL group. According to the current staging guideline, four patients with fewer than 16 retrieved LNs among node-positive patients were excluded, all of whom were in the non-FL group. When the perioperative clinicopathological features of the two groups were compared, there were significant differences in age (59.0 vs. 57.0 years, *P<*0.001), resection extent (*P<*0.001), clinical nodal stage (*P=*0.007), and histological differentiation (*P=*0.001) (Supplementary Table S1, Supplemental Digital Content 2, http://links.lww.com/JS9/A738). To minimize potential selection bias, after 1:1 propensity score matching, 1064 patients in each group were included in the final analysis (Fig. 1). The two groups were well-balanced in terms of preoperative tumour characteristics, clinical stage, resection, and lymphadenectomy extent (Table 1). All matched variables exhibited a standardized mean difference value of less than 0.1, and the generalized variance ratio was close to 1, demonstrating good balance between the groups.

**Table 1:** Clinical features in the fluorescent lymphography-guided lymphadenectomy group and the conventional lymphadenectomy group after propensity score matching.

Variable^*^	Non-FL group (*n*=1064), *N* (%)	FL group (*n*=1,064), *N* (%)	*P*	SMD	GVR
Age^a^, mean (SD), year	58.1 (12.4)	57.0 (12.4)	0.044	0.087	0.995
Sex^a^			0.402	0.038	1.014
Male	638 (60.0)	618 (58.1)			
Female	426 (40.0)	446 (41.9)			
BMI^a^, mean (SD), kg/m^2^	23.6 (3.1)	23.4 (3.1)	0.293	0.046	0.987
ASA score^a^			0.554	0.036	0.952
1	227 (21.3)	250 (23.5)			
2	594 (55.8)	587 (55.2)			
3	235 (22.1)	217 (20.4)			
4	8 (0.8)	10 (0.9)			
cT^a^			0.492	0.040	1.023
cT1	754 (70.9)	732 (68.8)			
cT2	218 (20.5)	247 (23.2)			
cT3	79 (7.4)	73 (6.9)			
cT4a	13 (1.2)	12 (1.1)			
cN^a^			0.662	0.037	1.074
cN0	91 (85.6)	897 (84.3)			
cN1	130 (12.2)	144 (13.5)			
cN2	23 (2.2)	23 (2.2)			
Tumour size^a^, mean (SD), mm	28.7 (20.6)	29.4 (20.8)	0.399	0.037	1.017
Location^a^			0.852	0.003	0.987
LC	361 (33.9)	360 (33.8)			
GC	203 (19.1)	204 (19.2)			
AW	215 (20.2)	216 (20.3)			
PW	277 (26.0)	280 (26.3)			
Circumferential	8 (0.8)	4 (0.4)			
Differentiation^a^			0.248	<0.001	0.922
Differentiated	400 (37.6)	387 (36.4)			
Undifferentiated	616 (57.9)	642 (60.3)			
Other	48 (4.8)	35 (3.3)			
Resection^a^			0.567	0.001	1.044
STG	841 (79.0)	852 (80.1)			
TG	151 (14.2)	135 (12.7)			
PG	72 (6.8)	77 (7.2)			
Dissection^a^			0.211	0.056	1.043
D1+	734 (69.0)	706 (66.4)			
D2	330 (31.0)	358 (33.6)			

a Matched variables.

χ^2^tests were used to evaluate categorical variables, and the Mann–Whitney U test was used for continuous variables.

ASA, American Society of Anesthesiologists; AW, anterior wall; FL, fluorescent lymphography-guided lymphadenectomy; GC, greater curvature; GVR, generalized variance ratio; LC, lesser curvature; non-FL, conventional lymphadenectomy; PG, proximal gastrectomy; PW, posterior wall; SMD, standardized mean difference; STG, subtotal gastrectomy; TG, total gastrectomy.

### LN retrieval and stage distribution

In the FL group, 59 804 LNs were retrieved from 11 214 LN stations. Of these, 49 670 (83.1%) were fluorescent LNs from 7368 (65.7%) fluorescent LN stations. The median number of retrieved LNs per patient was 53 [interquartile range (IQR), 42−67), including 44 (IQR, 32−57) fluorescent LNs per patient, corresponding to a median number of 10 (IQR, 9−11) dissected LN stations, including 7 (IQR, 5−8) fluorescent LN stations. In the non-FL group, 11 351 LN stations containing 49 150 LNs were dissected. The median number of retrieved LNs per patient was 44 (IQR, 34−56) in a median of 10 (IQR, 9−11) dissected LN stations.

The FL group showed significantly more retrieved LNs (mean, 56.2 vs. 46.2, *P<*0.001) (Table 2). The FL group also showed significantly more retrieved LNs in both LN-negative and LN-positive patients (54.2 vs. 45.3, *P<*0.001 and 63.0 vs. 49.8, *P<*0.001, respectively) than the non-FL group. Moreover, at least 30 LNs were retrieved from 1009 patients in the FL group (94.8%) and 890 patients in the non-FL group (83.6%, *P*<0.001). Postoperative complications, classified as Clavien–Dindo Grade III or higher, showed no significant difference between the two groups (4.8% vs. 3.6%, *P*=0.194). When comparing pathological nodal classifications, the number of LN-positive patients was 239 (22.5%) in the FL group and 205 (19.3%) in the non-FL group (*P=*0.078). In addition, the nodal classification of the FL group tended to be more advanced than the non-FL group, although there was no statistical difference (*P=*0.084) (Table 2). However, in accordance with these marginal nodal classification differences, the FL group showed significantly more advanced pathological stage distribution than the non-FL group (*P=*0.044). The proportion of stage I patients was reduced in the FL group (77.1%) compared with the non-FL group (81.4%), and the proportion of stage III patients increased (9.8% vs. 7.5%). As a result, these stage distribution differences affected the postoperative adjuvant chemotherapy proportions between the two groups. As the proportion of stage II and III increased in the FL group, 2.9% more patients in the FL group received adjuvant chemotherapy (19.5% vs. 16.6%, *P=*0.102), although it was not statistically significant.

**Table 2:** Pathological features in the fluorescent lymphography-guided lymphadenectomy group and the conventional lymphadenectomy group after propensity score matching.

Variable	Non-FL group (*n*=1064), *N* (%)	FL group (*n*=1064), *N* (%)	*P*
Retrieved LN, Mean (SD), No.	46.2 (18.2)	56.2 (20.1)	<0.001
≤15 (%)	16 (1.5)	1 (0.1)	<0.001
16–29	158 (14.8)	54 (5.1)	
≥30	890 (83.6)	1009 (94.8)	
No. metastatic LN, Mean (SD), No	0.8 (2.8)	1.2 (4.4)	0.012
^a^Complication grade III or higher			0.194
No	1013 (95.2)	1026 (96.4)	
Yes	51 (4.8)	38 (3.6)	
pT			0.126
pT1a	477 (44.8)	434 (40.8)	
pT1b	347 (32.6)	352 (33.1)	
pT2	99 (9.3)	100 (9.4)	
pT3	80 (7.5)	110 (10.3)	
pT4	61 (5.7)	68 (6.4)	
pN			
pN0	859 (80.7)	825 (77.5)	0.084
pN1	104 (9.8)	112 (10.5)	
pN2	62 (5.8)	64 (6.0)	
pN3	39 (3.7)	63 (5.9)	
Stage (AJCC 8^th^)			0.044
Stage I	866 (81.4)	820 (77.1)	
Stage II	118 (11.1)	140 (14.0)	
Stage III	80 (7.5)	104 (9.8)	
Adjuvant CTx			0.102
No	887 (83.4)	857 (80.5)	
Yes	177 (16.6)	207 (19.5)	

a Complication grade followed the Clavien–Dindo classification system.

AJCC, American Joint Committee on Cancer; CTx, chemotherapy; FL, fluorescent lymphography-guided lymphadenectomy; LN, lymph node; non-FL, conventional lymphadenectomy.

### Survival

Supplementary Fig. S1, Supplemental Digital Content 2, http://links.lww.com/JS9/A738 shows the overall and relapse-free survival of the entire FL and non-FL group before propensity score matching. Before matching, after a median follow-up duration of 41 months in both groups, the FL group showed higher overall survival than the non-FL group in all 3348 patients, although the difference was not statistically significant (*P=*0.057). Stratified by stage, the FL group showed higher overall survival than the non-FL group in stages II (*P=*0.045) and stage III (*P=*0.040), while no significant difference was found in stage I (*P=*0.200). However, relapse-free survival of the FL group was significantly higher than the non-FL group only in stage II (*P=*0.035). In contrast, no statistically significant differences were observed in relapse-free survival in stages I (*P=*0.160) and III (*P=*0.180).

**Figure 2:**
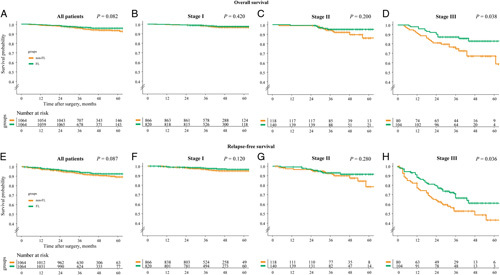
Kaplan–Meier survival curves between the fluorescent lymphography-guided lymphadenectomy group and the conventional lymphadenectomy group, comparing overall survival and relapse-free survival for all patients (A and E, respectively), stage I patients (B and F, respectively), stage II patients (C and G, respectively), and stage III patients (D and H, respectively).

After propensity score matching, both the overall and relapse-free survival in the FL group showed higher survival than in the non-FL group in all 2128 patients, although the difference was not statistically significant (Fig. 2) (*P=*0.082 and *P=*0.087, respectively). When stages were stratified, patients with stage I (*P*=0.420 and *P*=0.120, respectively) or stage II (*P*=0.200 and *P*=0.280, respectively) in the FL group showed similar overall and relapse-free survival to those in the non-FL group. However, patients with stage III in the FL group showed higher overall survival (*P*=0.038) and relapse-free survival (*P*=0.036) than those in the non-FL group.

## Discussion

This study showed that FL increased the retrieved LNs number and also the proportion of patients with 30 or more LNs. The increased retrieved LNs number by FL brought about more LN-positive patients. This resulted in more advanced stages due to a decrease in stage I patients and an increase in stage II or III patients when FL was performed. Furthermore, gastric cancer patients who underwent FL showed higher survival than those who underwent non-FL in stage III gastric cancer patients.

Fluorescent lymphography has gained attention in recent years for its potential to enable effective lymphadenectomy during gastric cancer surgery^27,28^. By clearly visualizing lymphatics in contrast to the surrounding tissue, it allows for thorough lymphadenectomy and intraoperative assessment of the lymphadenectomy adequacy. Fluorescent lymphography demonstrated high sensitivity and negative predictive value of fluorescent lymphography in detecting metastatic LNs^19^. Additionally, fluorescent lymphography increased the number of retrieved LNs, indicating effective lymphadenectomy^15–18,29,30^.

Adequate and thorough lymphadenectomy by identifying LNs stained with fluorescent resulted in a low rate of non-compliance, defined as the case where a specific LN station was dissected, but no LN was present^16,17^. The low non-compliance rate was associated with better locoregional cancer control and improved cancer-specific survival in gastric cancer surgery^6,31^. By dissecting the fluorescent-positive stations which contain LNs, the non-compliance rate can be reduced, which would improve cancer-specific survival. As well as the low non-compliance rate, the high sensitivity for detecting metastatic LNs indicates metastatic LNs would likely be present in the fluorescent-positive stations^19^. Thus, the high sensitivity and negative predictive value of FL in detecting metastatic LNs may reduce the probability of remaining metastatic LNs after lymphadenectomy. Improvement in locoregional control by removing all potential metastatic LNs and stations could reduce local recurrences.

Retrieving a larger number of LNs in gastric cancer surgery is associated with improved survival. Patients with more than 30 harvested lymph nodes showed better prognoses^10^. The FL group in this study showed an increased proportion of patients with 30 or more LN retrievals, indicating that fluorescent lymphography can contribute to obtaining the optimal number of LNs that can directly affect survival outcomes.

In this study, fluorescent lymphography revealed more LN-positive patients, resulting in more advanced stages due to a decrease in stage I patients and an increase in stage II or III patients. Harvesting a greater number of LNs caused an increase in nodal classifications, the Will Roger’s phenomenon, showing that the more LNs examined, the greater the LN metastasis^10^. With fluorescent lymphography, a larger proportion of patients were classified as LN-positive, resulting in higher proportions of stage II or higher patients, even with similar clinical characteristics to those undergoing non-FL.

Thus, changes in nodal stage distribution could have contributed to the higher proportion of stage III patients in the FL group. If patients who would have been diagnosed as stage II or lower with non-FL are classified as stage III due to the stage migration effect of fluorescent lymphadenectomy, they may have a better prognosis than non-FL stage III patients. The use of fluorescent lymphography enabled more accurate staging than non-FL and revealed a stage migration effect, which is critical for predicting patient prognosis by altering the stage distribution. Therefore, the FL group’s higher proportion of stage III patients with higher survival would be influenced by the stage migration effect, which led to the inclusion of patients with better prognoses with fluorescent lymphadenectomy.

Additionally, the stage migration effect associated with fluorescence lymphography could affect the proportion of patients receiving adjuvant chemotherapy proportion. The increase in stages II and III patients resulted in more frequent adjuvant chemotherapy following current guidelines after FL than after non-FL^7,8^. The more frequent adjuvant chemotherapy would influence the higher survival of gastric cancer patients after FL if a large number of advanced gastric cancer patients were included.

Overall, the use of fluorescent lymphadenectomy can have several positive prognostic effects, including better locoregional cancer control through thorough LN dissection, high sensitivity and negative predictive value for metastatic LN detection, and an increase in the percentage of patients with 30 or more retrieved LNs. Nevertheless, the stage migration effect was the essential cause of the higher survival seen in the stage III patients of the FL group, which included patients with a better prognosis than those in the non-FL group.

This study had some limitations, including its retrospective nature. The number of stage III patients included was relatively small. To confirm the effectiveness of FL in advanced gastric cancer patients, studies with larger numbers of advanced gastric cancer patients are necessary. This study could not evaluate whether dissections of fluorescent-positive stations only would have the same oncological outcomes as conventional systematic lymphadenectomy could not be evaluated. Therefore, to clarify the exact clinical efficacy and benefits of fluorescence lymphography in gastric cancer, a well-designed randomized controlled trial is warranted to compare conventional systematic lymphadenectomy with lymphadenectomy to dissect only fluorescent-positive stations. Additionally, the concentration and amount of ICG injection changed throughout the study period with the development of surgical equipment and robotic systems. However, there was no standardized optimization of ICG concentration for different platforms and systems. Given fluorescent imaging devices’ varying sensitivity and signal strength, future studies should be conducted to create optimized ICG injection quantities^32^. Lastly, we excluded patients who received neoadjuvant chemotherapy in this study due to potential lymphatic drainage pattern changes. The impact of fluorescent-guided lymphadenectomy after ICG injection on patients who have received neoadjuvant chemotherapy should be evaluated through further studies.

Despite these limitations, this study comprehensively assessed the long-term survival of gastric cancer patients after FL using a larger prospectively collected cohort. This study also showed the potential of FL to facilitate the more appropriate management of gastric cancer patients through more accurate staging compared with non-FL.

FL using near-infrared imaging in gastric cancer patients provided higher survival outcomes, particularly in stage III patients, due to more accurate staging resulting from stage migration, thorough lymphadenectomy, larger lymph node harvests, and high sensitivity in detecting metastatic lymph nodes. Thus, given its potential to improve prognostication by enhancing staging accuracy, it is recommended as an option to consider the use of FL in clinical practice.

## Ethical approval

This study was approved by the Institutional Review Board of Severance Hospital, Yonsei University Health System (4-2020-0082), which waived informed consent for the study because of its retrospective nature.

## Sources of funding

This study was supported by a grant from the Investigator Sponsored Research Program (ISR-2017-10924) and Covidien Private Limited (Medtronic). The funding sources played no role in the design of this study. They did not play any role in study execution, data analysis, data interpretation, or the decision to submit the results for presentation or publication.

## Author contribution

S.H.P.: study design, data acquisition, statistical analysis, writing, and manuscript revision. K.-Y.K.: data acquisition, statistical analysis. M.C.: data acquisition, interpreted the data. Y.M.K.: data acquisition, interpreted the data. H.-Il.K.: data acquisition, supervision. W.J.H.: study design, data acquisition, writing and manuscript revision, supervision.

## Conflicts of interest disclosure

W.J.H. received research grants from Medtronic and GC Pharma and is a stockholder and chief executive officer of Hutom. He provided consultancy services to Ethicon and SK Hynix outside the submitted work. All other investigators in this study have declared no potential conflicts of interest.

## Research registration unique identifying number (UIN)

1. Name of the registry: The Research Registry.
2. Unique Identifying number or registration ID: Researchregistry8817.
3. Hyperlink to your specific registration (must be publicly accessible and will be checked): https://www.researchregistry.com/browse-theregistry#home/registrationdetails/642d4845c21644002961d481/

## Guarantor

Woo Jin Hyung.

## Data statement

W.J.H. had full access to all data in the study and took responsibility for the integrity of the data and the accuracy of data analysis.

## Acknowledgements

The authors acknowledge the assistance of Bioscience Writers, LLC (Houston, TX, USA) with the editing and correcting of English language usage.

## Supplementary Material

SUPPLEMENTARY MATERIAL
