# Supplementary material for: Prognostic impact of fluorescent lymphography on gastric cancer
Source: Int J Surg. 2023 Jun 21;109(10):2926–33. doi: 10.1097/JS9.0000000000000572 (PMC10583912; doi:10.1097/JS9.0000000000000572)

Supplemental digital contents

Supplementary Table S1. Clinico-pathologic features in the fluorescent lymphography-guided lymphadenectomy group and the conventional lymphadenectomy group before propensity score matching

Supplementary Fig. S1. Kaplan-Meier survival curves between the fluorescent lymphography-guided lymphadenectomy group and the conventional lymphadenectomy group before propensity score matching, comparing overall survival and relapse-free survival for all patients (2A and 2E, respectively), stage I patients (2B and 2F, respectively), stage II patients (2C and 2G, respectively), and stage III patients (2D and 2H, respectively).

Supplementary Table S1. Clinico-pathologic features in the fluorescent lymphography-guided lymphadenectomy group and the conventional lymphadenectomy group before propensity score matching

Variable	Non-FL group (n = 2183), No. (%)	FL group (n = 1079), No. (%)	*P*-value	SMD	GVR
**Age, mean(SD), year**	59.0 (12.2)	57.0 (12.4)	<0.001	0.169	1.024
**Sex**			0.342	0.037	1.014
**Male**	1312 (60.1)	629 (58.3)			
**Female**	871 (39.9)	450 (41.7)			
**BMI, mean (SD), kg/m^2**	23.6 (3.1)	23.5 (3.1)	0.287	0.040	1.003
**ASA score**			0.342	0.046	0.941
**1**	459 (21.2)	255 (23.6)			
**2**	1203 (55.6)	594 (55.0)			
**3**	471 (21.8)	220 (20.4)			
**4**	30 (1.4)	11 (1.)			
**cT**			0.115	0.071	1.052
**cT1**	1578 (72.3)	742 (68.8)			
**cT2**	428 (19.6)	251 (23.3)			
**cT3**	151 (6.9)	74 (6.9)			
**cT4a**	26 (1.2)	12 (1.1)			
**cN**			0.007	0.110	1.261
**cN0**	1992 (88.0)	909 (84.2)			
**cN1**	232 (10.6)	146 (13.5)			
**cN2**	29 (1.3)	24 (2.2)			
**Tumor size, mean (SD), mm**	27.9 (19.5)	29.4 (20.8)	0.041	0.077	1.135
**Location**			0.079		
**LC**	845 (38.7)	365 (33.8)			
**GC**	381(17.5)	205 (19.0)			
**AW**	429 (19.7)	222 (20.6)			
**PW**	516 (23.6)	283 (26.2)			
**Circumferential**	12 (0.5)	4 (0.4)			
**Differentiation**			0.001	0.117	0.935
**Differentiated**	940 (43.1)	394 (36.5)			
**Undifferentiated**	1169 (53.6)	650 (60.2)			
**Other**	74 (3.4)	35 (3.2)			
**Resection**			<0.001	0.117	1.524

**STG**	1799 (82.4)	865 (80.2)			
**TG**	296 (13.6)	136 (12.6)			
**PG**	88 (4.0)	78 (7.2)			
**Dissection**			0.248	0.044	1.034
**D1+**	1496 (68.5)	717 (66.5)			
**D2**	687 (31.5)	362 (33.5)			
**Retrieved LN, Mean (SD), No.**	42.7 (17.2)	56.2 (20.1)	<0.001		
**≤15 (%)**	38 (1.7)	1 (0.1)	<0.001		
**16-29**	454 (20.8)	55 (5.1)			
**≥30**	1691 (77.5)	1023 (94.8)			
**No. metastatic LN, Mean (SD), No**	0.7 (2.6)	1.2 (4.4)	0.001		
**†Complication grade III or higher**			0.716		
**No**	2099 (96.2)	1041 (96.5)			
**Yes**	84 (3.8)	38 (3.5)			
**pT**			<0.001		
**pT1a**	966 (44.3)	439 (40.6)			
**pT1b**	775 (35.5)	359 (33.2)			
**pT2**	191 (8.7)	102 (9.4)			
**pT3**	141 (6.5)	112 (10.4)			
**pT4**	110 (5.)	68 (6.3)			
**pN**			0.001		
**pN0**	1805 (82.7)	837 (77.6)			
**pN1**	199 (9.1)	111 (10.3)			
**pN2**	104 (4.8)	65 (6.0)			
**pN3**	75 (3.4)	67 (6.2)			
**Stage (AJCC 8th)**			<0.001		
**Stage I**	1822 (83.5)	831 (77.0)			
**Stage II**	221 (10.1)	141 (13.1)			
**Stage III**	140 (6.4)	107 (9.9)			
**Adjuvant CTx**			<0.001		
**No**	1865 (85.4)	869 (80.5)			
**Yes**	318 (14.6)	210 (19.5)			

Abbreviations: non-FL, conventional lymphadenectomy; FL, fluorescent lymphography-guided lymphadenectomy; SMD, standardized mean difference; GVR, generalized variance ratio; SD, standard deviation; BMI, body mass index; ASA, American Society of

Anesthesiologists; LC, lesser curvature; GC, greater curvature; AW, anterior wall; PW, posterior wall; STG, subtotal gastrectomy; TG, total gastrectomy; PG, proximal gastrectomy; LN, lymph node; AJCC, American Joint Committee on Cancer; CTx, chemotherapy

†Complication grade followed the Clavien-Dindo classification system

Supplementary Fig. S1. Kaplan-Meier survival curves between the fluorescent lymphography-guided lymphadenectomy group and the conventional lymphadenectomy group before propensity score matching, comparing overall survival and relapse-free survival for all patients (2A and 2E, respectively), stage I patients (2B and 2F, respectively), stage II patients (2C and 2G, respectively), and stage III patients (2D and 2H, respectively).

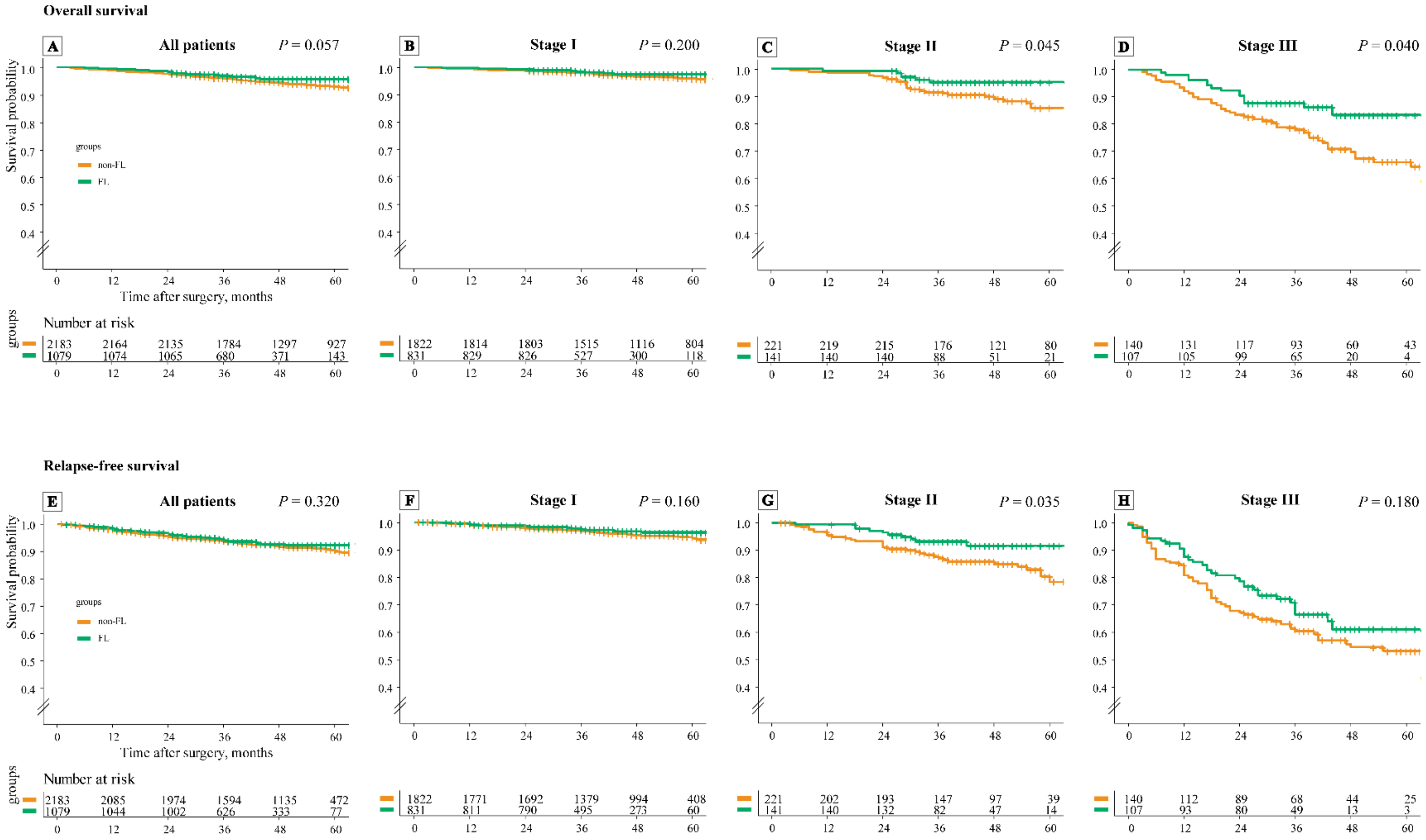

Supplement: SUPPLEMENTARY MATERIAL [file js9-109-2926-s002.pdf]
